# House Dust Mite Nebulization Drives Alarmin and Complement Activation in a Murine Tracheal Air–Liquid Interface Culture System

**DOI:** 10.3390/cells14201598

**Published:** 2025-10-14

**Authors:** Janti Haj Ahmad, Philip Einwohlt, Mareike Ohms, Doris Wilflingseder, Jörg Köhl

**Affiliations:** 1 Institute for Systemic Inflammation Research, University of Lübeck, 23562 Lübeck, Germany; janti.hajahmad@uksh.de (J.H.A.); philip.einwohlt@uksh.de (P.E.); mareike.ohms@uksh.de (M.O.); 2 Ignaz Semmelweis Institute, Interuniversity Institute for Infection Research, Vetmeduni, 1210 Vienna, Austria

**Keywords:** innate immunity, alarmins, air-liquid interface, airway epithelium, house dust mite, complement system, C3a, C5a, C3aR, C5aR1

## Abstract

Air–liquid interface (ALI) cultures offer a physiologically relevant in vitro model of the airway epithelium (AE), capable of recapitulating key structural and functional features observed in vivo. In this study, we established and validated a murine ALI culture system comprising pseudostratified epithelia with functional tight junctions, ciliated cells and goblet cells. To assess their innate immune functions, we designed and 3D-printed an autoclavable aerosol deposition chamber, which allowed us to expose differentiated AE cultures to house dust mite (HDM) allergen. Upon HDM exposure, AE cells mounted a time-dependent innate immune response characterized by the secretion of complement component C3, the generation of its active cleavage products C3a and increased expression of C3aR and C5aR1. This was associated with increased intracellular TSLP and IL-25 production and TSLP release in AE cells. Progressive loss of tight junction integrity and reduced transepithelial electrical resistance (TEER) demonstrated epithelial susceptibility to allergen protease-induced cell damage. Together, we established a murine ALI system preserving airway epithelial architecture and a nebulization system to study innate immune activation of AE cells in response to HDM mimicking the initial phase of allergen sensitization. More generally, we described a powerful and accessible platform for studying epithelial-driven mechanisms in murine airway immune responses.

## 1. Introduction

The mucosal surface of the AE serves as the primary interface between the external environment and the respiratory system, providing a mechanical and immunological barrier against airborne particulate matter, allergens and pathogens. Composed of multiple specialized cell types, including ciliated cells, goblet cells and tight-junction-forming epithelial cells, such cells synergize to maintain airway integrity and sterility [1,2]. The AE can be primed and activated by pathogens, allergens or pathogen-associated molecular patterns (PAMPs) that can bind and stimulate the AE. It is this versatile immune activity of the AE in sensing and clearing pathogens, allergens and PAMPs that maintains its integrity [3,4]. Several cell culture models have been developed to represent different segments of the respiratory system, from the nasal cavity down to the alveolar space. The complexity of the in vitro models ranges from submerged monocultures [5,6,7] to advanced multi-cell-type cultures at ALI [8,9].

Several studies reported that exposure to inhaled air pollutant particulate can precipitate the development of chronic lung disease, such as chronic obstructive lung disease, asthma and chronic bronchitis [10,11]. Cell culture models that mimic the structural and functional properties of the AE are indispensable for studying respiratory immunity, disease mechanisms and potential therapies. Among these, ALI cultures have emerged as a powerful platform for modeling mucociliary differentiation, mucus production, epithelial barrier function and innate immune responses in vitro [12,13,14]. Coupled with genetically modified murine models, ALI cultures can provide unique insights into how the innate or adaptive immune system regulates airway epithelial responses to pathogens [15,16] or allergens [17,18]. Previously, we and others have shown that the complement system is activated in the lungs of patients suffering from allergic asthma [19,20] and in a mouse model of HDM-induced pulmonary allergy [21,22,23]. Further, several reports suggest that C3a and C5a contribute to allergen sensitization through an impact on dendritic and innate lymphoid type 2 (ILC2) cells [21,22,24,25]. Given that complement factors and receptors are expressed in AE cells [26] and can control epithelial cell function [27,28,29], we considered murine ALI cultures an excellent model system to explore the contribution of AE-derived complement factors and their receptors to allergen sensitization.

Traditionally, ALI cultures have been stimulated either by submerging the cultures in a diluent containing the stimulant or by using a handheld sprayer to apply the stimulant to the surface of the cultures. However, both methods have significant limitations. Submerging mature ALI cultures disrupts the integrity of the epithelial barrier and alters the transcriptomic state [30]. On the other hand, using a sprayer lacks precision in dosage delivery, often resulting in inconsistent and uneven exposure of the culture surface. Pharmaceutical sprayers commonly face the issue of variable dose delivery. The positioning of the sprayer bottle can significantly affect the volume dispensed from such sprayers, resulting in up to 75-fold increase in volume delivery [31]. These drawbacks underscore the need for a more refined approach.

Here, we designed an exposure chamber to nebulize the potent aeroallergen HDM onto the apical surface of ALI cultures, simulating physiologically relevant allergen exposure. To enhance experimental reproducibility, we developed a custom data logger for the Millicell-ERS2 system, enabling precise and consistent monitoring of TEER values during culture propagation and following allergen exposure. Further, we used such tools and methods to validate murine ALI cultures as a robust and versatile platform to determine the impact of HDM exposure on complement activation and alarmin production in the AE. This approach allowed us to perform a detailed analysis of complement system activation, barrier integrity and epithelial cell modulation in response to aeroallergen exposure.

## 2. Materials and Methods

### 2.1. Mice

C57BL/6J mice were bred and kept in the specific pathogen-free facility of the University of Lübeck according to the institutional and national guidelines. The euthanization of mice for organ removal was approved by the Ministry of Agriculture, Energy Transition and Rural Areas, Kiel, Germany, according to the Animal Welfare Act §4, paragraph 3 (AZ 39_2021-03-29_Köhl and 39_2024-06-25_Köhl), and it was performed by certified personnel. The mice used were 7–17 weeks old of both sexes.

### 2.2. Media and Buffers

PneumaCult^TM^-ExPlus (STEMCELL Technologies #05040—Vancouver, BC, Canada) and PneumaCult^TM^-ALI-S (STEMCELL Technologies #05001—Vancouver, BC, Canada) were used as per the manufacturer specifications. The isolation media were composed of DMEM/F12 (Gibco (Thermo Fisher Scientific) #11330032—Grand Island, NY, USA) supplemented with 100 U/mL Penicillin and 100 µg/mL Streptomycin (Gibco (Thermo Fisher Scientific) #15140122—Grand Island, NY, USA) and 2.5 µg/mL Amphotericin B (Gibco (Thermo Fisher Scientific) #15290018—Grand Island, NY, USA). The dissociation media comprised DMEM/F12 supplemented with 100 U/mL Penicillin and 100 µg/mL Streptomycin, 2.5 µg/mL Amphotericin B and 0.15% pronase (Roche #10165913103—Basel, Switzerland). The expansion media were composed of KSFM (Gibco (Thermo Fisher Scientific) #17005042—Grand Island, NY, USA) supplemented with 100 U/mL Penicillin and 100 µg/mL Streptomycin, 0.025 µg/mL murine EGF (Peprotech #315-09—Cranbury, NJ, USA), 0.03 mg/mL bovine pituitary extract (BPE) (Gibco (Thermo Fisher Scientific) #13028014—Grand Island, NY, USA), 1 µM Isoproterenol (Sigma-Aldrich #I-6504—Burlington, MA, USA), 10 µM fresh Y-27632 (STEMCELL Technologies #72304—Vancouver, BC, Canada) and 5 µM fresh DAPT (STEMCELL Technologies #72082—Vancouver, BC, Canada). The proliferation and differentiation media shared common basic media prepared using DMEM/F12 supplemented with 100 U/mL Penicillin and 100 µg/mL Streptomycin, 0.03% (*w*/*v*) NaHCO_3_ (Gibco (Thermo Fisher Scientific) #25080094—Grand Island, NY, USA) and 1.5 mM L-Glutamine (Gibco (Thermo Fisher Scientific) #25030-024—Grand Island, NY, USA). The proliferation media were prepared using basic media, 5% FBS (Capricorn #FBS-ES-HI-12A—Ebsdorfergrund, Germany), 1× fresh insulin–transferrin–selenium (ITS-G) (Gibco (Thermo Fisher Scientific) #41400045—Grand Island, NY, USA), 0.025 µg/mL murine EGF, 0.03 mg/mL BPE, 0.05 µM retinoic acid (RA) (Miltenyi Biotec #130-117-339—Bergisch Gladbach, Germany), 0.1 µg/mL fresh cholera toxin (Sigma-Aldrich #C8052—Burlington, MA, USA), 10 µM fresh Y-27632 and 5 µM fresh DAPT. The differentiation media were prepared using basic media, 0.1% FBS, 1× fresh ITS-G, 0.005 µg/mL murine EGF, 0.03 mg/mL BPE, 0.05 µM RA and 0.025 µg/mL fresh cholera toxin. The block/perm solution was prepared using 1% BSA (Sigma-Aldrich #A9418-100G—Burlington, MA, USA), 1% donkey serum (Jackson ImmunoResearch #AB_2337258—West Grove, PA, USA) and BD perm/wash (BD Biosciences #554723—San Jose, CA, USA) in PBS. This solution was used to permeabilize ALI cultures after fixation with BD Cytofix (BD Biosciences #554655—San Jose, CA, USA). The intracellular block—performed to reduce non-specific binding of antibodies to intracellular components—was achieved using the same block/perm solution. The solution was also used for diluting both primary and secondary antibodies.

### 2.3. Murine Tracheal Isolation and Culture

C57BL/6J wildtype mice were euthanized via CO_2_ asphyxiation. The tracheae were removed and cut from the thyroid cartilage down to the tracheobronchial junction, stripped from the attached tissue, cut open lengthways and placed in a 50 mL tube (Sarstedt #62.547.254—Nümbrecht, Germany) containing 10 mL isolation media supplemented with 15 mg of pronase and incubated overnight at 4 °C. The next day, 10 mL of the isolation media containing 20% FBS was added to the 50 mL falcon tube, and the tube was gently inverted ~20 times. Next, a 100 µm cell strainer (Sarstedt #83.3945.100—Nümbrecht, Germany) was placed on top of a new 50 mL falcon tube, and the content of the 50 mL falcon tube was pipetted through the cell strainer to separate the trachea and large debris from the tracheal cells. The 50 mL falcon tube containing the tracheal cells was then centrifuged at 400× *g* for 10 min. The supernatant (SN) was discarded, and 1 mL of sterile isolation media supplemented with 0.5 mg of DNAse I (Sigma-Aldrich #DN25-10MG—Burlington, MA, USA) and 1 mg of BSA was used to resuspend the tracheal cell pellet and incubated for 5 min on ice. Then, the cells were centrifuged at 350× *g* for 5 min, followed by discarding the SN and resuspending tracheal cells in the expansion media.

### 2.4. Expansion of Murine Tracheal Cells

Isolated tracheal cells were seeded on cell culture flasks coated with 0.2% cold water fish gelatine (Sigma-Aldrich #935425-500MG—Burlington, MA, USA). The coating was performed by adding 0.2% cold water fish gelatine to the culture flask and incubating it at 37 °C for 30 min at 5% CO_2_. The gelatine solution was removed by pipetting, and the flask was washed once with 37 °C sterile PBS. Once the cells were seeded, 10 mL of the expansion media was added to the T-75 culture flask (Sarstedt #83.3911.002—Nümbrecht, Germany) and placed in a cell culture incubator at 37 °C with 5% CO_2_ and media change every 48 h. The cells were expanded to 80–90% confluency before being harvested for seeding onto tissue culture (TC) inserts (Sarstedt #83.3932.101—Nümbrecht, Germany) to start the ALI culture.

### 2.5. Harvesting Confluent Tracheal Cells and Propagation on TC Inserts

Confluent tracheal cells were harvested by removing the expansion media and submersing the cells in TrypLE express enzyme (Gibco (Thermo Fisher Scientific) #12605010—Grand Island, NY, USA) for 20–30 min. The cells were counted, collected in a 50 mL falcon tube and centrifuged at 400× *g* for 10 min at RT. The SN was discarded, and the cell pellet was resuspended in KFSM propagation media. In a 24-well plate (Sarstedt #83.3922.500—Nümbrecht, Germany), a TC insert was placed into each well. The basal compartment was filled with 600 µL of propagation media. The apical compartment was seeded with 8 × 10^4^ harvested tracheal cells, and the volume was topped with propagation media to 200 µL. TEER measurements were performed, including a blank TC insert to record day 0 TEER values. KFSM propagation media were changed every 48 h.

### 2.6. TEER Measurements and Logging Data

TEER values were measured using an STX01 probe (Merck #MERSSTX01—Darmstadt, Germany) connected to Millicell ERS-2 (MERCK #MERS00002—Darmstadt, Germany). The Millicell ERS-2 device was calibrated using the provided 1 kΩ resistor. To measure TEER values, the apical and basal compartments contained 200 µL or 600 µL of PBS (Gibco (Thermo Fisher Scientific) #14190169—Grand Island, NY, USA) at RT. The longer end of STX01 was placed into the basal compartment, while the shorter end was placed into the apical compartment. Since TEER values are temperature-sensitive, ALI cultures were allowed to equilibrate to room temperature (RT) for 10 min. Values were recorded using the data logger connected to the analog output of the Millicell ERS-2. The custom data logger averages two hundred readings per second from Millicell ERS-2 and logs the average of the reading on a text document that can be downloaded by connecting to a Wi-Fi access point created by the data logger (Document S1 and File S1).

### 2.7. Differentiation of Confluent Tracheal Cells on TC Inserts

Once the propagating cells on TC inserts were confluent and the TEER measurements were >2 kΩ·cm^2^, the propagating cells were airlifted, and the differentiation process started. The airlift process was conducted by removing the propagation media from the basal and apical compartments and replacing them with differentiation media in the basal compartment, leaving the apical compartment exposed to air. Cultures were allowed 10–14 days to differentiate and develop cilia. A thin mucus film could be noticed on the air-exposed apical side of the TC insert, which was removed weekly. The differentiation media were changed every 48 h.

### 2.8. Nebulization Chamber Design and 3D Printing

**Figure 1:**
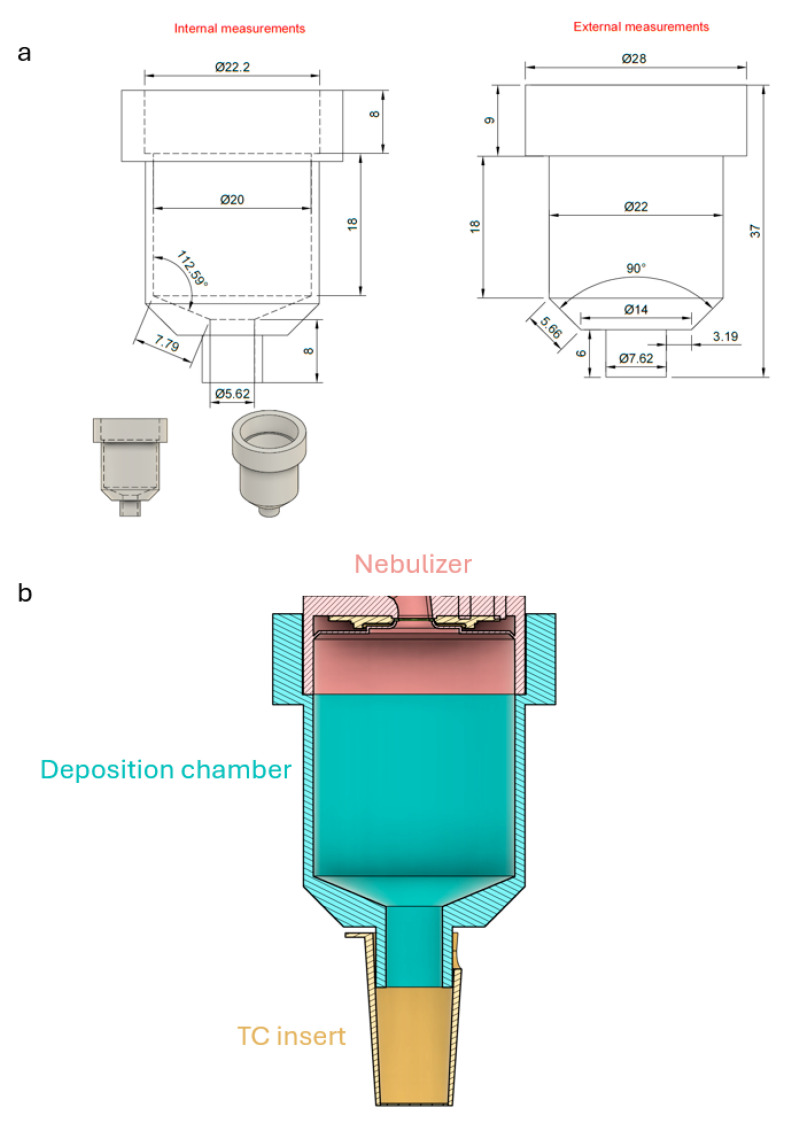
Design and implementation of the 3D-printed deposition chamber. (**a**) The 3D model of the deposition chamber was designed in Autodesk Fusion 360, inspired by a previous report [32]. The model was manufactured using additive manufacturing with the Prusa SL1S MSLA printer and Bio-Med Clear biocompatible resin. Post processing was performed according to the manufacturer’s instructions. The measurements are indicated in mm. (**b**) The assembled deposition chamber fits the Aerogen Pro nebulizer output at the wider end and connects precisely to the apical side of a 24-well TC insert at the narrower end. The design ensures friction fit without rubber O-rings to prevent aerosol leakage.

The 3D model of the deposition chamber was inspired by a previous report [32] and designed using the Autodesk Fusion 360 software (Autodesk GmbH, Munich, Germany) (Figure 1a). The model was then implemented into a device through additive manufacturing using 3D-printing technology. The Prusa SL1S MSLA printer (Prusa Research, Prague, Czech Republic) provided by FabLab Lübeck e. V. (Lübeck, Germany) was used to print the chamber with 3D-printable resin, i.e., the autoclavable Bio-Med Clear biocompatible resin (Liqcreate #LBMC01000, Utrecht, The Netherlands). The deposition chamber model was sliced using the PrusaSlicer (Prusa Research, Prague, Czech Republic). Post processing and curing of the printed chamber were performed following Liqcreate’s recommendations. The wider opening of the deposition chamber fits the output of the Aerogen Pro nebulizer unit (Inspiration Medical GmbH #AG-AP1000—Würzburg, Germany) connected to an Aerogen USB controller (Inspiration Medical GmbH #AG-UC1000-NE—Würzburg, Germany); by comparison, the smaller opening precisely connects to the apical opening of a 24-well TC insert (Sarstedt #83.3932.101—Nümbrecht, Germany) (Figure 1b). Further, it serves as a friction fit without using rubber O-rings to prevent leakage of nebulized aerosols. The nebulization chamber design files and 3D files can be found in the Supplementary Files (File S2).

### 2.9. Determination of Nebulization Chamber Deposition Efficiency and Stimulation of ALI Cultures with HDM in 24-Well Plates

For initial testing of the deposition efficiency of the 3D-printed deposition chamber, fluorescein sodium salt (Th. Geyer GmbH #46960-25G-F—Renningen, Germany), diluted in sterile H_2_O, was nebulized. First, the Aerogen Pro nebulizer unit was connected to the wider opening of the 3D-printed deposition chamber. Next, the smaller opening of the deposition chamber was inserted into the apical opening of a TC insert. A volume of 50 µL of fluorescein sodium salt solution was added to the nebulizer mesh, and the device was turned on until the entire volume was nebulized, a process that took ~8 s. Three different concentrations were tested, i.e., 50 μg/mL, 150 μg/mL and 250 μg/mL. The resulting mist was allowed to settle for either 10 s, 30 s or 60 s. Once the time had lapsed, the TC insert and 3D-printed chamber were separated, and the outlet of the Aerogen Pro nebulizer unit and 3D chamber were washed with PBS between each nebulization to remove residual liquid. The apical compartment of the TC insert was washed with 100 µL of sterile H_2_O and placed in a 96-well plate. The deposited mass of fluorescein sodium was measured using the FLUOstar Omega microtiter plate reader (BMG Labtech, Ortenberg, Germany) at 485 nm excitation and 520 nm emission wavelength against a standard curve. To calculate the deposition efficiency, the following equation was used:

$$\left(deposited\;mass\;in\;µg÷nebulized\;mass\;in\;µg\right)\;\times 100=deposition\;ef\;ficiency$$

For stimulation of ALI cultures, 50 µL of 1.5 mg/mL of HDM (Greer #XB70D3A25—Lenoir, NC, USA) was nebulized and allowed to settle for 30 s on day 13 ALI cultures in differentiation media. The chamber was cleaned in between nebulization to remove residual fluids.

### 2.10. Determination of Anaphylatoxin Receptor and Epithelial Cell Marker Expression

ALI cultures were washed three times with PBS at 37 °C to remove residual differentiation media. A volume of 200 µL of BD Cytofix (BD Bioscience #554655—San Jose, CA, USA) fixation buffer was added to the apical compartment of the TC insert and incubated for 10 min at RT. The fixation buffer was removed, and the cells were washed three times with PBS. A volume of 200 µL of block/perm solution was added to the apical compartment of the TC inserts of ALI cultures for 20 min. Primary antibodies directed against MUC5AC (Abcam #ab212636—Cambridge, UK) were conjugated to Alexa Fluor (AF) 488 (Abcam #ab236553—Cambridge, UK); C5 (clone BB5.1, Hycult Biotech #HM1073—Uden, The Netherlands) was conjugated to AF 594 (Abcam #ab269822—Cambridge, UK); acetyl-alpha tubulin (Thermo Scientific #32-2700—Waltham, MA, USA) was coupled with rabbit anti-mouse FITC (Dako #F0261—Santa Clara, CA, USA) as a secondary antibody; beta IV tubulin (Abcam #ab11315—Cambridge, UK) was coupled with rabbit anti-mouse FITC (Dako #F0261—Santa Clara, CA, USA) as a secondary antibody; ZO-1 (Invitrogen (Thermo Fisher Scientific) #61-7300—Waltham, MA, USA) was coupled with donkey anti-rabbit AF 594 (Jackson Immuno Research #711-585-152—West Grove, PA, USA) as a secondary antibody; C5aR1 (Hycult Biotech #HM10076—Uden, The Netherlands) was coupled with donkey anti-rat AF 594 (Invitrogen (Thermo Fisher Scientific) #A21209—Waltham, MA, USA) as a secondary antibody; and C3aR (HycultBiotech #HM1123—Uden, The Netherlands) was coupled with donkey anti-rat AF 488 (Invitrogen (Thermo Fisher Scientific) #A21208—Waltham, MA, USA) as a secondary antibody. Primary antibodies were diluted in the block/perm buffer and incubated overnight at 4 °C. The next day, primary antibodies were removed, and ALI cultures were washed three times with the block/perm buffer. Secondary antibodies were diluted in the block/perm buffer and incubated at RT for 1 h, followed by three washes with the block/perm buffer. Finally, Hoechst 33342 (Thermo Scientific #62249—Waltham, MA, USA) was used for nuclear staining for 20 min at RT, followed by three washes with the block/perm solution. The TC insert membrane was removed by gently cutting the membrane out using a scalpel. The membrane was then mounted using Fluoroshield (Sigma-Aldrich #F6182-20mL—Burlington, MA, USA). Images were acquired as Z-stacks for full-thickness imaging of the ALI TC insert using the Keyence BZ-X800 fluorescence microscope (Keyence Corporation, Osaka, Japan).

Z-stack immunofluorescence images were processed in ImageJ 1.54J (NIH—Bethesda, MD, USA) using the Sum projection function to generate composite images for each field of view. These projection images were then imported into CellProfiler v4.2.6 (Available at: https://cellprofiler.org/) for quantitative analysis. Images were first converted from RGB to grayscale using the “ColorToGray” module to separate individual fluorescence channels. Each channel was then processed independently using the “IdentifyPrimaryObjects” module, excluding objects located at the edges of the image.

For object identification, a manual threshold of 0.1 was applied, with a smoothing scale set to 1.3488. Clumped objects were distinguished based on intensity, and dividing lines were drawn using the “propagate” method. Following object identification, fluorescence intensities were quantified using the “MeasureObjectIntensity” module. Output data were exported as spreadsheets and subsequently analyzed. Lastly, we used VHX-7000 digital microscope (Keyence Corporation, Osaka, Japan) to capture the movement of cilia on video from a day 7 ALI culture. Operation of the VHX-7000 was carried out by a trained Keyence employee. The captured video (Video S1) is the raw format with no editing or processing.

### 2.11. Assessment of C3, C3a, C5, C5a and Alarmin Expression

The quantification of complement components and anaphylatoxins was performed using both in-house and commercial ELISA methods. All samples were assayed in technical duplicates, and absorbance was read at 450 nm with wavelength correction at 540 nm using a microplate reader (FLUOstar Omega, BMG LABTECH, Ortenberg, Germany).

A custom sandwich ELISA was developed to detect mouse C5. High-binding 96-well plates (Corning #9018—Corning, NY, USA) were coated overnight at 4 °C with 50 µL/well of 2 µg/mL anti-mouse C5 capture antibody (clone BB5.1, Hycult Biotech #HM1073—Uden, Netherlands). Plates were sealed with ELISA plate sealers (R&D Systems #DY992—Minneapolis, MN, USA). The next day, plates were washed four times with 0.05% Tween 20 in PBS and blocked for 90 min at RT with 200 µL of 2% BSA in PBS. Plates were washed four times with 0.05% Tween 20 in PBS; serial dilutions of recombinant mouse C5 standard (Acro Biosystems #CO5-M52H4—Newark, DE, USA) were performed; and samples were added (25 µL/well) and incubated at RT for 90 min. After washing four times with 0.05% Tween 20 in PBS, 25 µL/well of 1 µg/mL in 1% BSA/PBS biotinylated anti-mouse C5a detection antibody (clone I52-278, BD Biosciences #558028—San Jose, CA, USA) was added and incubated for 90 min at RT. After washing the plates four times with 0.05% Tween 20 in PBS, 50 µL/well Streptavidin-HRP (Thermo Scientific #N504—Waltham, MA, USA), diluted 1:1000 in PBS, was applied for 30 min with shaking. Plates were washed four times with 0.05% Tween 20 in PBS followed by 100 µL/well TMB substrate (Thermo Scientific #34028—Waltham, MA, USA) for 20 min. The reaction was stopped with 1 M H_2_SO_4_, and absorbance was measured immediately.

Another custom ELISA was performed to detect mouse C3a. Plates were coated with 25 µL/well of 4 µg/mL purified anti-mouse C3a capture antibody (clone I87-1162, BD Biosciences #558250—San Jose, CA, USA) in PBS and incubated overnight at 4 °C. Plates were washed four times with PBS containing 0.05% Tween 20, then blocked for 1 h at RT with 200 µL of 2% BSA in PBS. Plates were washed four times with 0.05% Tween 20 in PBS; serial dilutions of recombinant mouse C3a (R&D Systems #8085-C3-025—Minneapolis, MN, USA) were performed; and samples were added (25 µL/well) and incubated at RT for 90 min. After washing four times with 0.05% Tween 20 in PBS, 25 µL/well of 1 µg/mL in 1% BSA/PBS biotinylated anti-mouse C3a detection antibody (clone I87-419, BD Biosciences #558251—San Jose, CA, USA) was added and incubated for 1 h at RT. After washing the plates four times with 0.05% Tween 20 in PBS, 50 µL/well Streptavidin-HRP (Thermo Scientific #N504—Waltham, MA, USA), diluted 1:1000 in PBS, was applied for 30 min with shaking. Plates were washed four times with 0.05% Tween 20 in PBS followed by 100 µL/well TMB substrate (Thermo Scientific #34028—Waltham, MA, USA) for 20 min. The reaction was stopped with 1 M H_2_SO_4_, and absorbance was measured immediately.

Mouse C5a levels were measured using a commercial ELISA kit (R&D Systems, #DY2150—Minneapolis, MN, USA); C3 concentrations were quantified using a kit from Hycult Biotech (HycultBiotech #HK2002—Uden, The Netherlands); alarmin concentrations were quantified using commercial ELISA kits (R&D systems, TSLP #DY555, IL-33 #DY3626, IL-25/IL-17E #DY1399—Minneapolis, MN, USA). The manufacturers’ protocols were followed.

ELISA data were normalized via a correction factor to account for differences in AE cell numbers on the TC inserts. To calculate the correction factor, we first determined the protein concentrations in the lysates and SN from unstimulated control cells and from all cells stimulated for 24, 48 or 72 h with HDM. Then, we calculated the mean of the protein concentrations obtained from the cell lysates and SN. This mean value was then divided by the protein concentration determined in the individual samples to calculate the correction factor, which was used to multiply the raw values determined by ELISA to obtain the normalized value. The protein concentrations in the cell lysate and in the SN were assessed by Pierce Rapid Gold BCA protein Assay Kit (Thermo Scientific #A53225—Waltham, MA, USA), following the manufacturer’s protocol.

### 2.12. Assessment of C3 Cleavage to C3a by HDM

To assess the functional cleavage of C3 and generation of C3a by HDM, we incubated 1 µg of serum-purified hC3 [33] in DPBS for 30 min at 37 °C with 1 µg/mL of HDM (Greer #XB70D3A25—Lenoir, NC, USA) or HDM, which was heat-inactivated at 65 °C for 30 min (HI-HDM). After the incubation with HDM or HI-HDM, a cocktail of protease inhibitors (Thermo Scientific #78430—Waltham, MA, USA) was added to stop the protease activity of HDM. To determine the generation of hC3a, we performed a hC3a neo-epitope-specific ELISA, as described [34]. Briefly, we used neo-epitope-specific anti-hC3a mAb (Hycult Biotech #HM2074—Uden, The Netherlands) as capture and biotinylated anti-hC3/C3a mAb (Hycult Biotech #HM2073—Uden, The Netherlands) as detection antibody. After the addition of Streptavidin-HRP (Thermo Scientific #N504—Waltham, MA, USA), plates were developed with TMB substrate (Thermo Scientific #34028—Waltham, MA, USA) for 20 min. The reaction was stopped with 1 M H_2_SO_4_, and absorbance was measured immediately.

### 2.13. Statistical Analysis

For statistical evaluation, the GraphPad PRISM 10 (San Diego, CA, USA) software was used. Data are represented as mean ± standard error of the mean (SEM). Prior to hypothesis testing, data were assessed for normality using the Shapiro–Wilk test. If the assumption of normality was violated, non-parametric analyses were performed using the Kruskal–Wallis test, followed by Dunn’s multiple comparisons test. If data were normally distributed, one-way analysis of variance (ANOVA) was used, followed by Holm–Šidák’s multiple comparisons test. The concentrations of complement components, anaphylatoxins and alarmins measured by ELISA were analyzed using two-way ANOVA followed by Dunnett’s multiple comparisons test, comparing each stimulated condition to the unstimulated control, or Tukey’s multiple comparisons test, comparing all groups to each other. HDM’s ability to cleave C3 was assessed using a two-tailed unpaired *T*-test. Differences between groups were considered significant at * *p* < 0.05, ** *p* < 0.01, *** *p* < 0.001 and **** *p* < 0.0001.

## 3. Results

### 3.1. Basal Cell Expansion, Propagation and Functional Differentiation of ALI Cultures

**Figure 2:**
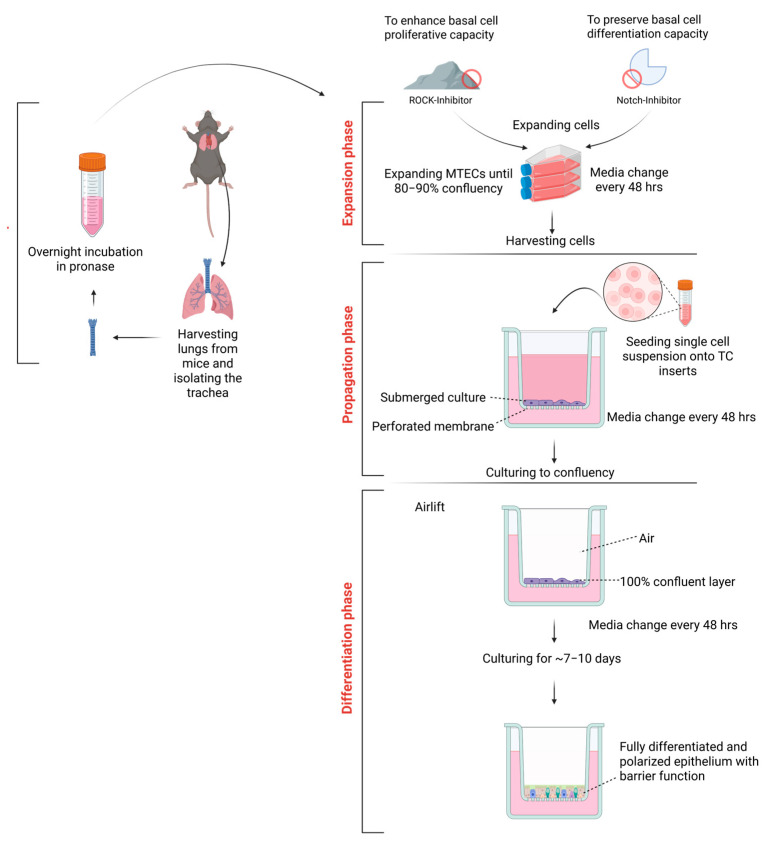
Workflow for the isolation, expansion and differentiation of MTECs. Harvesting phase: Murine tracheae were isolated from C57BL/6J mice following euthanasia, dissected from the thyroid cartilage to the tracheobronchial junction and enzymatically digested overnight in pronase-containing isolation media at 4 °C (Harvest phase). Expansion phase: Cells were seeded onto culture flasks pre-coated with 0.2% cold water fish gelatine and expanded at 37 °C with 5% CO_2_ using expansion media until 80–90% confluency. Propagation phase: Confluent cells were enzymatically detached, counted, centrifuged and resuspended in propagation media for seeding onto 24-well TC inserts. TEER was measured to assess barrier integrity and monitor confluency. Differentiation phase: Once cells reached confluency and TEER values exceeded 2 kΩ·cm^2^, ALI culture was initiated by removing the apical media and replacing basal media with differentiation media, leaving the apical surface exposed to air. Created in BioRender. Haj Ahmad, J. (2025) (https://BioRender.com/esgi47j).

First, we sought to generate high numbers of morphologically and physiologically differentiated murine tracheal epithelial cells (MTECs) characterized by tight junction formation, ciliated cells and goblet cells. For this purpose, we aimed to keep the cell renewal and differentiation potency of basal epithelial cells—the progenitor cells of the mouse trachea [35]—intact. We obtained 2 × 10^5^ cells per trachea, which is in accordance with previously recorded numbers [36,37]. This number allowed us to seed ~two TC inserts per mouse. Our goal was to increase this number by expanding the isolated MTECs ex vivo to obtain sufficient cells to run multiple experiments with fewer mice (Figure 2).

**Figure 3:**
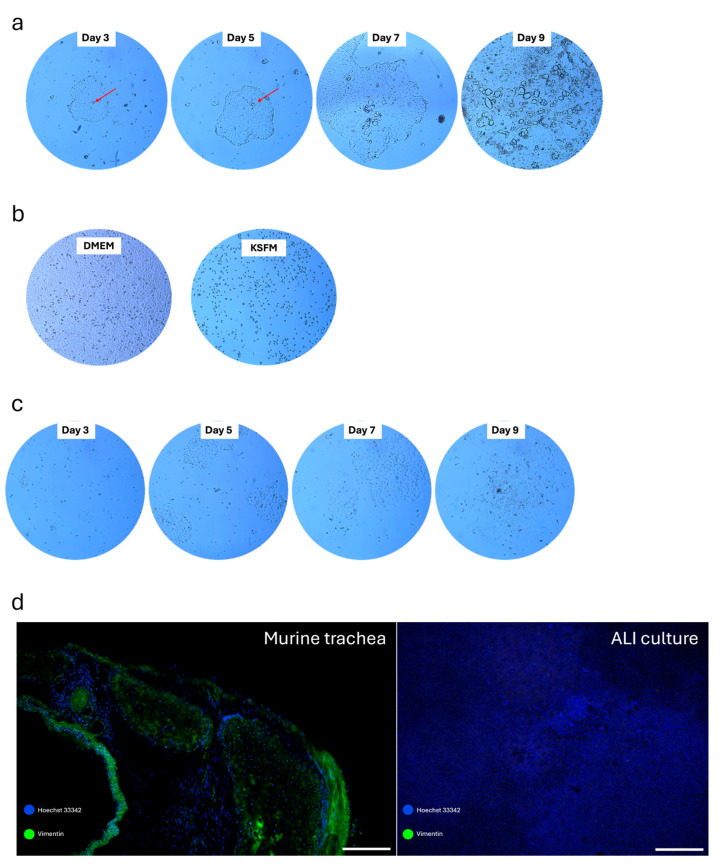
MTEC expansion and elimination of contaminating fibroblasts. (**a**) Tracheal cells cultured in PneumaCult™-Ex Plus Medium showed poor expansion, intracellular vacuolation and loss of differentiation capacity by day 9 of culture. The red arrows shown in the cultures from day 3 and day 5 point to the developing intracellular vacuoles. (**b**) To assess the fibroblast-supporting properties of expansion media, murine fibroblasts were cultured in either DMEM or supplemented KSFM media. Fibroblasts in DMEM reached confluency with typical morphology, while those in KSFM were shrunken, weakly adherent and failed to proliferate. (**c**) Improved tracheal cell expansion and morphology, preventing the formation of intracellular vacuoles and swollen cells after addition of Y-27632 and DAPT to KSFM-based expansion media. The magnification of the pictures shown in (**a**–**c**) is 40-fold. (**d**) Absence of vimentin-expressing fibroblasts in the tracheal cell population expanded in KSFM medium after 30 days in culture (7 days of expansion, 10 days of propagation, 13 days of differentiation). Scale bar = 20 µm.

Thus, we cultured the murine tracheal cells in commercially available media PneumaCult™-Ex Plus Medium designed for the expansion of primary human airway cells. The successful expansion of murine tracheal cells using such media has been demonstrated [38,39,40,41]. When we used PneumaCult™-Ex Plus Medium, we observed poor expansion, swollen intracellular vacuoles and failure to propagate and differentiate the tracheal cells after 9 days of culture (Figure 3a). To improve the differentiation process, we used KFSM media supplemented with EGF, BPI and isoproterenol, which have been successfully used to culture human [42,43] and murine epithelial cells [44]. Further, Notch targeting was found to increase the number of basal cells in ALI cultures [45,46,47], while the selective Rho-associated protein kinase inhibitor Y-27632 has been shown to enhance basal cell proliferative capacity without affecting the differentiation properties [48]. Given that Notch is a key target of γ-secretase, and DAPT is a potent inhibitor of the γ-secretase complex, we further supplemented the KFSM medium with DAPT and Y-27632. As fibroblasts tend to overgrow other cells and are considered a contaminant in primary cell cultures [49], we first tested the potency of fibroblasts to grow in the supplemented KFSM medium and compared it to DMEM. Fibroblasts cultured in DMEM reached full confluency and maintained their morphology, while those cultured in KSFM media were shrunken, poorly adherent and failed to proliferate (Figure 3b). The addition of Y-27632 and DAPT to the expansion media of tracheal cells resulted in their successful expansion and subsequent passaging in line with previous findings [44]. Morphologically, tracheal cells in Y-27632 and DAPT-supplemented media showed no intracellular vacuoles or swollen cells (Figure 3c). Finally, we tested the elimination of fibroblasts from murine trachea cells after expansion in KSFM media by staining for vimentin, which is primarily expressed in mesenchymal cells, such as fibroblasts. No vimentin signal from KSFM-expanded trachea cells was observed after 30 days (7 days of expansion, 10 days of propagation and 13 days of differentiation) (Figure 3d).

**Figure 4:**
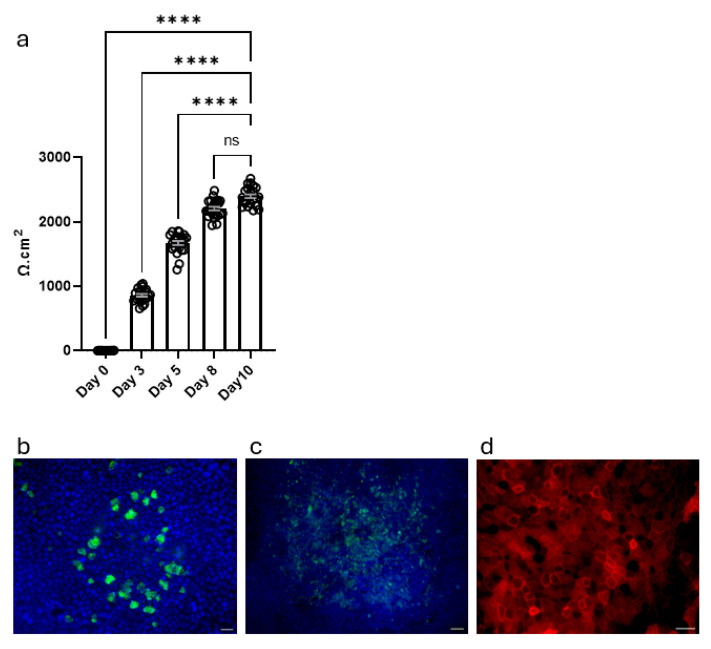
Barrier formation and mucociliary differentiation of MTECs on TC inserts. (**a**) TEER values of MTECs over 10-day differentiation. TEER values progressively increased, plateauing around day 8, indicating tight junction formation. Upon reaching TEER values ≥ 2 kΩ·cm^2^, cultures were airlifted, and the differentiation phase started. Data are shown as mean ± SEM (*n* = 23 separate 24-well inserts monitored from seeding (Day 0) to start of differentiation (Day 10)). Data were analyzed using Kruskal–Wallis test with Dunn’s multiple comparisons test. **** *p* < 0.0001. (**b**) Immunostaining for β-tubulin IV (green: FITC) shows the presence of well-developed ciliated cells on the apical surface, indicating successful onset of ciliary differentiation. (**c**) MUC5AC staining (green: AF-488) depicts mucus-producing goblet cells, confirming the establishment of a mucus-secreting epithelial phenotype. (**d**) ZO-1 staining (red: AF-594) shows continuous and well-defined tight junctions at cell borders, supporting the TEER data and indicating an intact epithelial barrier. Scale bar = 20 µm.

Successfully expanded tracheal cells (8 × 10^4^) were harvested and seeded onto 24-well TC inserts and propagated to confluency in KFSM medium (Figure 2). During this phase, we tested the ability of the cells to form a pseudostratified epithelial layer and exert a barrier function through tight junction formation. For this purpose, we determined TEER values from day 3 to day 10. They increased from day 0 to day 8 and reached a plateau by day 8 (Figure 4a). In addition to the two-hundred-readings-per-second ability of the custom data logger, its ability to store values in a text document allowed us to measure multiple wells and record their values in a short time, saving the operator’s time and maintaining consistency. Once the TEER values were at 2 kΩ·cm^2^, the KFSM propagation medium in the apical and basal compartments was removed and replaced by KFSM differentiation medium lacking Y-27632 or DAPT in the basal compartment, while the apical compartment remained exposed to air. The ALI cultures were then differentiated for 7 days and stained for cilia (beta IV tubulin), as this time point is characterized by the onset of ciliary motility [50], goblet cell (MUC5AC) and tight junction (ZO-1) markers (Figure 4b–d). Anti-beta IV tubulin staining showed well-developed ciliated cells on the apical surface, confirming successful mucociliary differentiation following 7 days of ALI culture (Figure 4b). Additionally, we were able to capture the cilia movement on a VHX-7000 digital microscope, demonstrating that the ALI cultures differentiated into ciliated cells with functional cilia (Video S1). Further, MUC5AC staining highlighted mucus production by goblet cells in differentiated ALI cultures, demonstrating a functional mucus-secreting environment (Figure 4c). Finally, ZO-1 staining confirmed the TEER measurements and revealed well-defined and continuous junctional structures between borders of epithelial cells, suggesting intact epithelial barrier formation (Figure 4d). Of note, we were able to maintain ALI cultures for up to 40 days without loss of barrier integrity.

### 3.2. Impact of Aerosol Concentration and Settling Time on Deposition Efficiency

**Figure 5:**
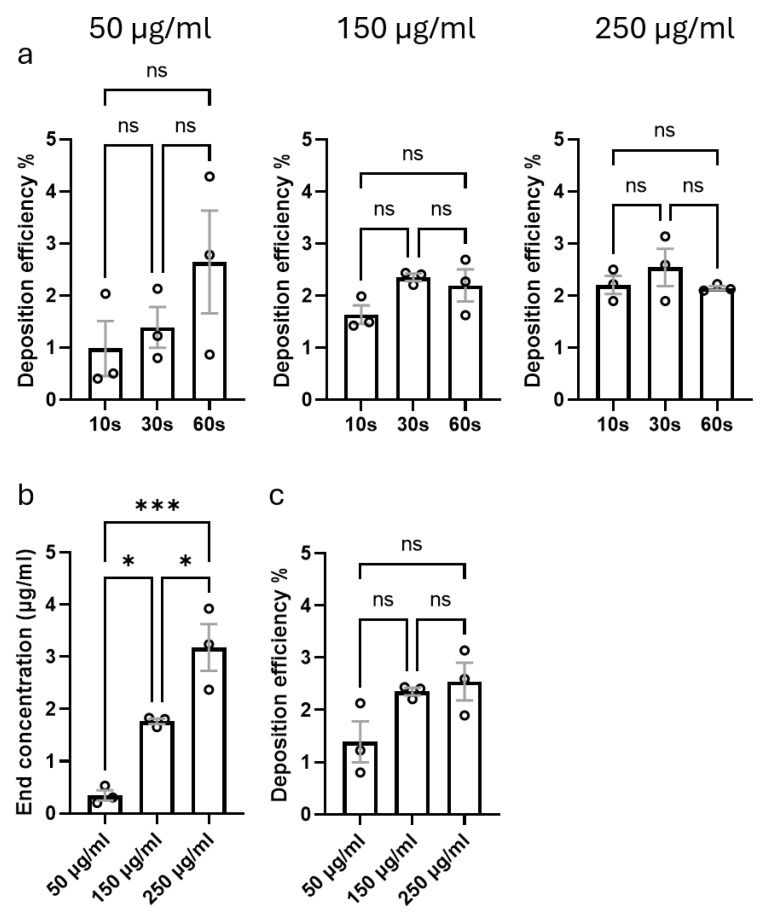
Effect of aerosol concentration and settling time on deposition efficiency of fluorescein sodium. (**a**) A volume of 50 µL of fluorescein sodium at three concentrations (50 µg/mL, 150 µg/mL and 250 µg/mL) was nebulized, and deposition was assessed after settling time of 10, 30 or 60 s. (**b**) Impact of increasing concentrations of nebulized fluorescein sodium on the deposition efficiency. (**c**) Impact of aerosol concentrations on the overall deposition efficiency defined as the ratio of recovered to applied fluorescein. Data are shown as mean ± SEM of three independent nebulization experiments. Data were analyzed using one-way ANOVA with Holm–Šidák’s multiple comparisons test. * *p* < 0.05; *** *p* < 0.001.

To efficiently nebulize aeroallergens, we used the commercially available Aerogen Pro nebulizer unit together with an Aerogen USB controller, which was connected to a custom-made 3D-printed deposition device (Figure 1a). The device was placed on the apical opening of a 24-well TC insert. In this setting, the deposition chamber does not touch the bottom of the insert, forming a closed unit together with the TC insert (Figure 1b). To assess the impact of aerosol concentration and settling times on deposition efficiency, we nebulized 50 µL of three different concentrations of fluorescein sodium, i.e., 50 μg/mL, 150 μg/mL and 250 μg/mL, which were allowed to settle for either 10, 30 or 60 s. First, we noticed that settling time did not affect the deposition efficiency, regardless of the concentration of the aerosol (Figure 5a). Further, while the increased aerosol concentration resulted in a higher end concentration of the fluorescein sodium in the plate (Figure 5b), the deposition efficiency was independent of the aerosol concentration (Figure 5c). These data show that the chamber allows controlled dosage delivery, ensuring reproducibility and mimicking physiological exposure conditions.

### 3.3. Assessment of C3 and C5 Production, the Generation of Anaphylatoxins C3a and C5a and the Expression of Their Receptors in Response to HDM Nebulization in ALI Cultures

The complement C3- and C5-derived anaphylatoxins C3a and C5a are known to contribute to the development of allergic asthma through their impact on dendritic cells and ILC2 [51]. Further, the anaphylatoxins facilitate leukocyte recruitment and activation, enhance vascular permeability and induce mast cell degranulation and smooth muscle contraction [52]. However, while some reports have shown expression of C3 in airway epithelial cells and highlighted its functional role in infection [28,29], less is known about the expression of C3a, C3aR, as well as C5, C5a and C5aR1 in the AE under steady-state conditions and in response to aeroallergens [26]. To better understand the regulation and dynamics of complement activation in the AE, we assessed the expression of C3 and C5 and the generation of their cleavage products, C3a and C5a, as well as the expression of their receptors, C3aR and C5aR1, in mature ALI cultures under steady-state conditions and in response to HDM exposure using our custom 3D deposition chamber. To investigate complement activation dynamics under different stimulation times, we quantified C3, C3a, C5 and C5a levels in cell lysates and SN using ELISA. We also assessed C3aR and C5aR1 median signal intensity via immunofluorescence across unstimulated cultures and cultures subjected to 24, 48 and 72 h of HDM nebulization.

**Figure 6:**
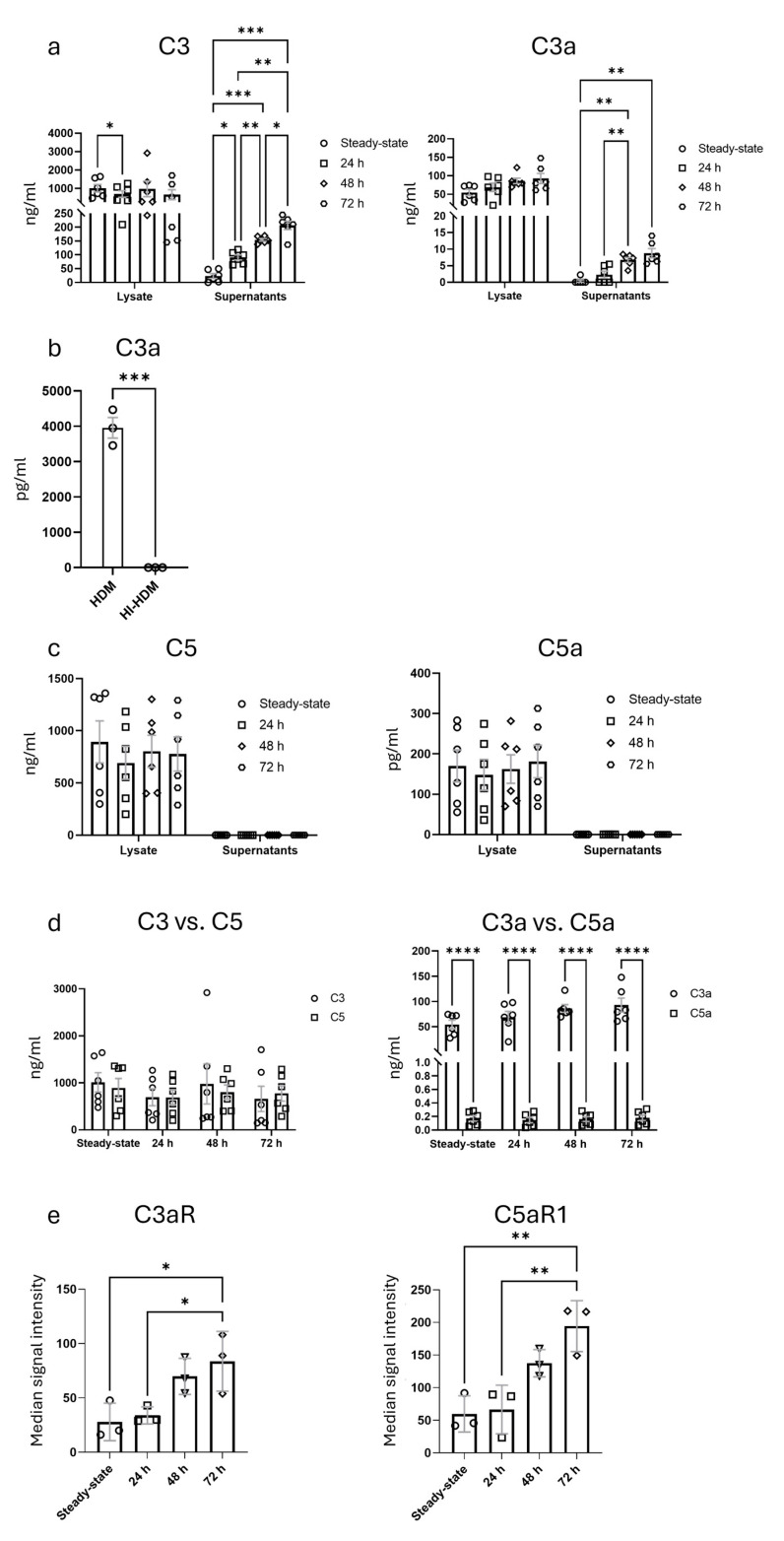
Time-dependent complement activation and anaphylatoxin receptor expression in ALI cultures exposed to HDM. (**a**) C3 and C3a concentrations in cell lysates and the SN of unstimulated (steady-state) ALI cultures and 24, 48 and 72 h after HDM exposure (steady-state, 24, 48 and 72 h: *n* = 6). * *p* < 0.05; ** *p* < 0.01; *** *p* < 0.001. (**b**) HDM and HI-HDM mediated cleavage of hC3 into hC3a (*n* = 3 independent experiments). Data are shown as mean ± SEM. Data were analyzed using an unpaired *T*-test. *** *p* < 0.001. (**c**) C5 and C5a concentrations in cell lysates and the SN of steady-state ALI cultures and 24, 48 and 72 h after HDM exposure. Data are shown as mean ± SEM (steady-state, 24, 48 and 72 h: *n* = 6). (**d**) Comparison of C3 and C5 (**left panel**), C3a and C5a (**right panel**) concentrations in cell lysates from steady-state ALI cultures and 24, 48 and 72 h after HDM exposure. Data are shown as mean ± SEM (steady-state, 24, 48 and 72 h: *n* = 6). Data were analyzed using two-way ANOVA with Šidák’s multiple comparisons test. **** *p* < 0.0001. (**e**) Immunofluorescence analysis of C3aR and C5aR1 expression in unstimulated ALI cultures and 24, 48 and 72 h after HDM exposure. Data are shown as mean ± SEM (*n* = 3). Data were analyzed using one-way ANOVA with Holm–Šidák’s multiple comparisons test. * *p* < 0.05; ** *p* < 0.01. The number of experiments shown in (**a**,**c**–**e**) refers to biological replicates derived from 6 independent preparations of trachea, each representing a separate cell isolation and culture.

We found consistently higher C3 and C3a concentrations in cell lysates as compared to the corresponding supernatants, showing dominant intracellular localization (Figure 6a). In lysates, C3 levels dropped during the initial 24 h following HDM exposure from 1010 ± 221 ng/mL in unstimulated cultures to 695 ± 182 ng/mL. During the next 24 h, the C3 levels increased to 977 ± 394 ng/mL, followed by a drop to 661 ± 280 ng/mL at 72 h. Intracellular C3a levels showed a time-dependent increase from 55 ± 7 ng/mL to 93 ± 15 ng/mL 72 h after HDM exposure, suggesting intracellular cleavage of C3 (Figure 6a). C3 concentration in the SN increased almost 9-fold over time from 23 ± 9 ng/mL in unstimulated cultures to 207 ± 15 ng/mL 72 h after HDM exposure, demonstrating a strong C3 release in response to HDM. Further, we observed a significant increase in C3a levels in the SN over time, which were highest 72 h after HDM exposure (8.8 ± 1.4 ng/mL), suggesting C3a secretion and/or C3a generation from secreted C3. As HDM exerts strong serine protease activity, we tested the ability of HDM to cleave C3 into C3a. As shown in Figure 6b, HDM cleaved C3 to C3a, while heat-inactivated HDM (HI-HDM) had no effect, suggesting that serine proteases in HDM drive C3a generation from secreted C3.

Similar to C3 and C3a, we observed dominant C5 expression and C5a generation in cell lysates as compared to the SN (Figure 6c). Intracellular C5 concentrations were comparable to intracellular C3 concentrations, ranging from 893.1 ± 201.3 ng/mL in unstimulated cultures to 692 ± 186 ng/mL 24 h after HDM stimulation (Figure 6d). In contrast to C3 and C3a, we found no C5 or C5a in the SN (Figure 6c), suggesting either no release, rapid degradation or, in the case of C5a, sequestration via binding to C5aR1. Of note, intracellular C3a levels were ~300-fold higher than C5a under steady-state conditions and ~500-fold higher 72 h after HDM exposure (Figure 6d, right panel), suggesting strong intracellular cleavage of C3 but not C5.

**Figure 7:**
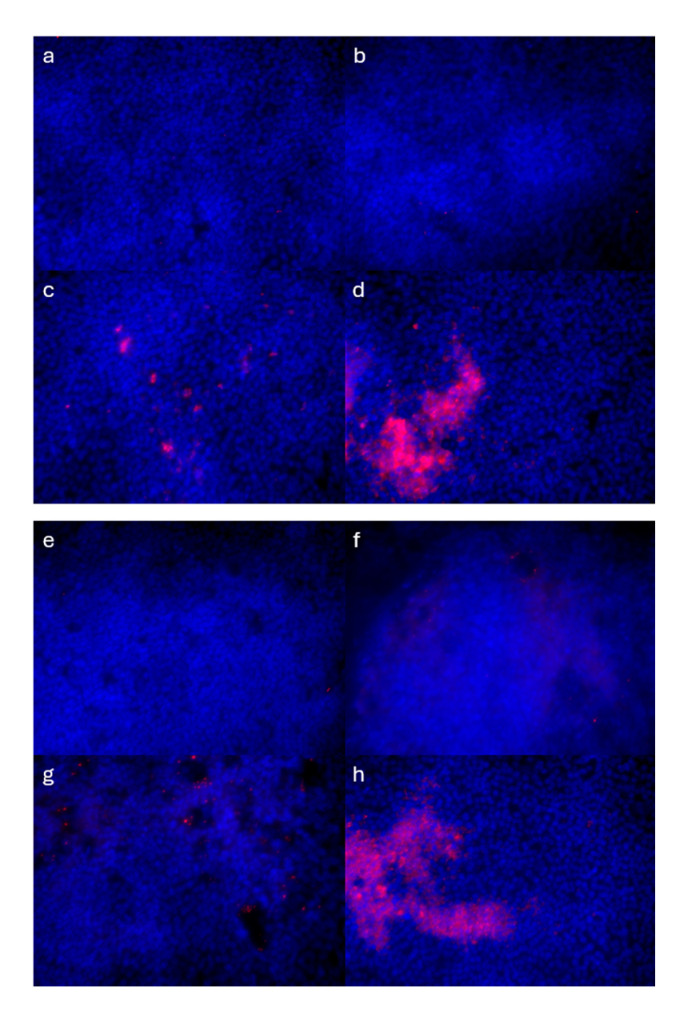

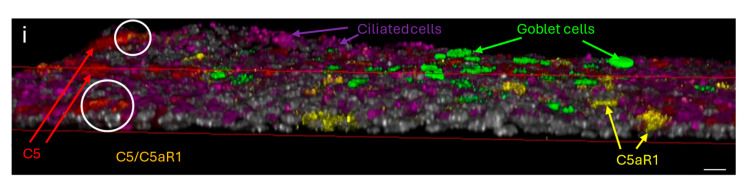
C3aR, C5 and C5aR1 expression in ALI cultures before and after HDM stimulation. (**a**–**d**) C5aR1 expression (red) in (**a**) unstimulated ALI culture and (**b**) 24 h, (**c**) 48 h or (**d**) 72 h after HDM exposure. (**e**–**h**) C3aR expression (red) in (**e**) unstimulated ALI culture and (**f**) 24 h, (**g**) 48 h or (**h**) 72 h after HDM exposure. (**i**) Z-stack 3D reconstruction of ALI culture showcasing the heterogeneity of AE cells in unstimulated ALI culture and the patchy expression of C5 and C5aR1. Ciliated cells (purple: acetylated tubulin), goblet cells (green: MUC5AC), C5 (red), C5aR1 (yellow); white circles point toward overlap of C5 and C5aR1 expression (orange). Nuclear stain is in gray (Hoechst 33342). Red arrows point toward C5 staining; yellow arrows indicate C5aR1 staining; and green arrows point toward goblet cells. The two red lines seen horizontally are part of the “view box” from the Leica microscope software (LAS X 4.8.1.29271—Wetzlar, Germany). Scale bar = 50 µm.

Immunofluorescence analysis of C3aR and C5aR1 expression revealed a significant and time-dependent upregulation correlated with the duration of HDM exposure (Figure 6e and Figure 7a–h). Further, we observed co-expression of C5 and C5aR1 in some AE cells (Figure 7i). Collectively, these findings demonstrate that complement activation under the tested conditions is predominantly intracellular, with minimal free extracellular presence. Furthermore, the observed time-dependent upregulation of C3aR and C5aR1 suggests an adaptive response to sustained complement activity. Importantly, these results demonstrate that ALI cultures are responsive to HDM exposure, supporting their utility as a model system for studying airway complement response.

### 3.4. Repeated HDM Exposure Results in Impaired Tight Junction Organization

**Figure 8:**
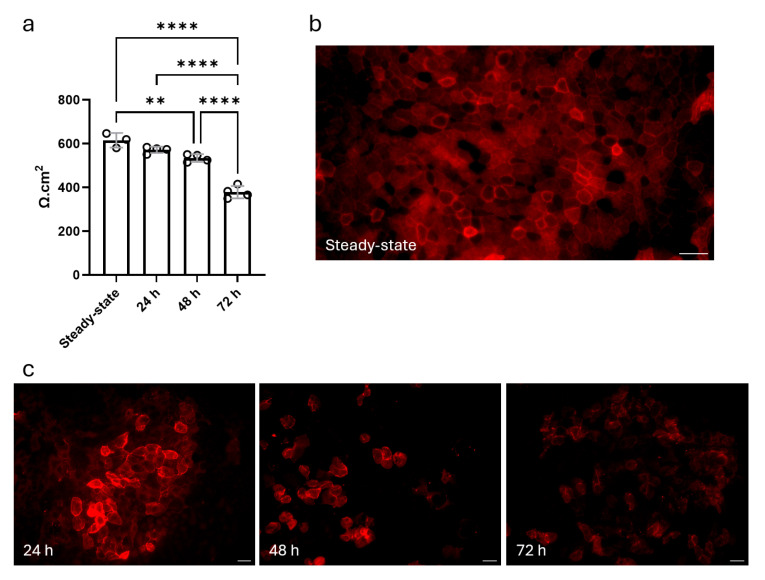
HDM exposure compromises epithelial barrier integrity in a time-dependent manner. (**a**) TEER measurements showed a progressive decline over 24, 48 and 72 h of HDM exposure, indicating reduced epithelial barrier function. Data are shown as mean ± SEM (steady-state: *n* = 3; 24, 48 and 72 h: *n* = 4; the number of experiments refers to biological replicates derived from 3 or 4 independent preparations of tracheas, each representing a separate cell isolation and culture). Data were analyzed using one-way ANOVA with Holm–Šidák’s multiple comparisons test. ** *p* < 0.01; **** *p* < 0.0001. (**b**) ZO-1 immunostaining showing continuous tight junction localization between epithelial cells in steady-state controls. (**c**) Disruption of ZO-1 organization over time, with fragmented and diffuse staining in response to HDM exposure. Scale bar = 20 µm.

Next, we assessed the impact of HDM exposure on epithelial cell integrity. We found that TEER values decreased steadily after HDM exposure, suggesting a progressive compromise of epithelial barrier function, particularly after 72 h (Figure 8a). Also, tight junction protein ZO-1 IF staining revealed notable changes in tight junction organization. In PBS-control ALI cultures, ZO-1 was continuously localized between epithelial cells, demonstrating intact tight junctions (Figure 8b). After 24 h of HDM exposure, ZO-1 staining appeared less continuous, with areas of fragmentation and disorganization emerging. These disruptions became more pronounced at 48 h and 72 h of exposure, with diffuse ZO-1 staining and gaps in junctional integrity (Figure 8c).

### 3.5. ALI Cultures Mimic In Vivo Airway Epithelium Activation in Response to HDM Exposure

**Figure 9:**
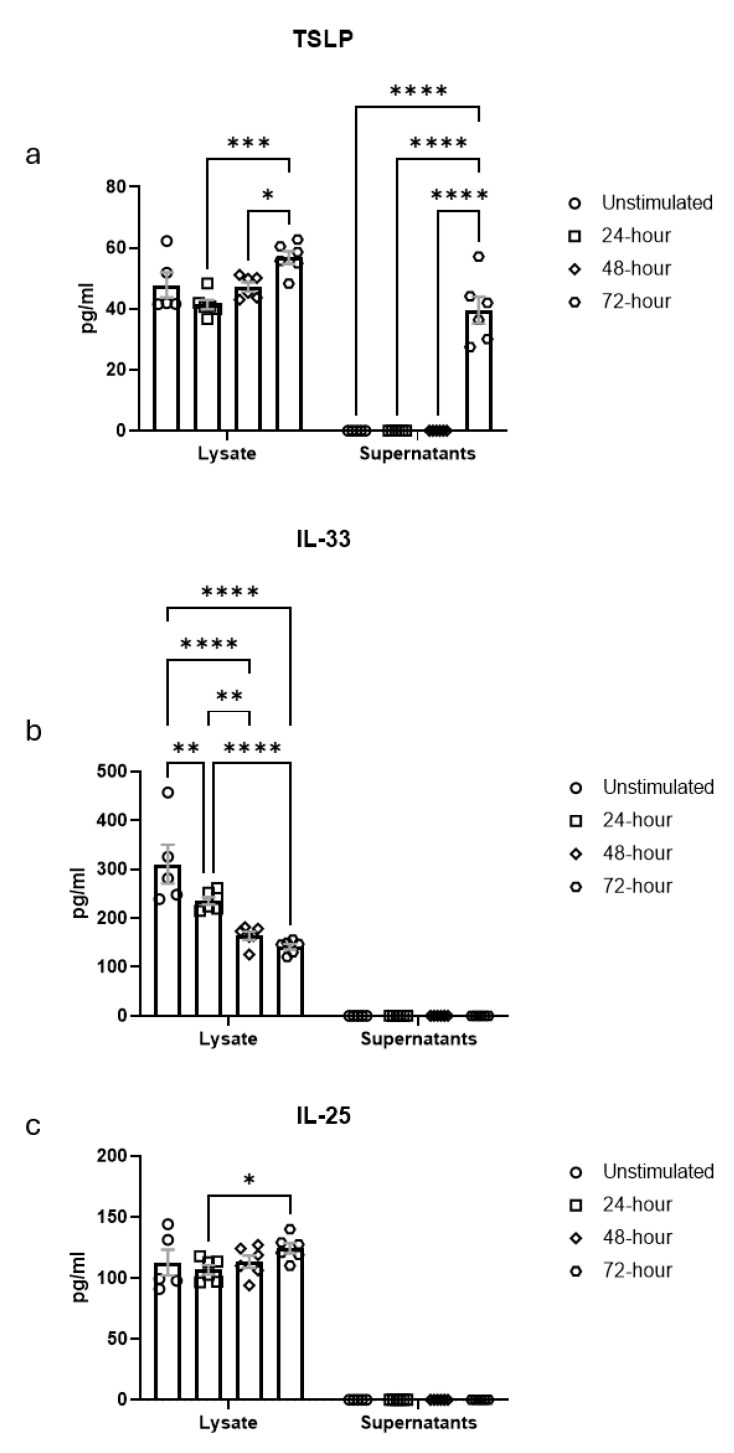
Impact of repeated HDM exposure on the induction of epithelial-derived alarmins in ALI cultures. (**a**–**c**) TSLP (**a**), IL-33 (**b**) and IL-25 (**c**) concentrations in cell lysates (**left panel**) and the SN (**right panel**). Data are shown as mean ± SEM (steady-state: *n* = 5; 24, 48 and 72 h: *n* = 6 biological replicates derived from 6 independent tracheal preparations, each representing a separate cell isolation and culture). Data were analyzed using two-way ANOVA with Tukey’s multiple comparisons test. * *p* < 0.05; ** *p* < 0.01; *** *p* < 0.001; **** *p* < 0.0001.

Finally, we determined the impact of HDM challenge on alarmin production of AE cells. We stimulated ALI cultures with HDM for 24, 48 and 72 h. The SN and cell lysates were collected at each time point, including unstimulated controls, and tested for levels of thymic stromal lymphopoietin (TSLP), IL-33 and IL-25 using ELISA. In the unstimulated epithelial cells, we found an exclusive expression of all alarmins within epithelial cells. The IL-33 concentrations (310 ± 89 pg/mL) were ~3-fold higher than IL-25 levels (113 ± 23 pg/mL) and ~6.5-fold higher than TSLP concentrations (48 ± 9 pg/mL) (Figure 9a–c). While intracellular IL-33 levels steadily declined during the first 48 h after HDM nebulization (Figure 9b), TSLP (Figure 9a) and IL-25 (Figure 9c) concentrations increased 72 h after HDM exposure. TSLP was the only alarmin that was secreted into the SN in significant amounts 72 h after HDM exposure (Figure 9a), whereas IL-33 and IL-25 concentrations were at the level of the detection limit of the assays (Figure 9b,c). IL-33 might have undergone rapid oxidation and formation of disulfide bridges following its release.

## 4. Discussion

In this study, we describe a method for strongly expanding primary MTECs after one passage in vitro and their subsequent ALI differentiation into ciliated and secretory cells in a pseudostratified epithelial cell layer. In the past, different types of media and supplements were successfully used to propagate and differentiate murine ALI cultures [53,54,55]. These studies described the methods for isolating and culturing mouse airway epithelial cells, with notable similarities and differences in their steps and media formulations. Antunes et al. [53] and Brockman-Schneider et al. [54] share similarities in several key steps, including enzymatic dissociation of epithelial cells using pronase and DNase, followed by differential adherence or centrifugation to remove fibroblasts. Both also involve initial culture in DMEM/F12 supplemented with FBS and antibiotics to support epithelial cell growth. In contrast, Eenjes et al. [44] differ in their approach, particularly by using KSFM expansion medium, which is formulated to prevent fibroblast growth without the need for pre-adherence steps, and distinct proliferation and differentiation media. Despite these differences, all three methods include steps for culturing cells at an ALI to promote differentiation and cilia formation, demonstrating a shared goal of optimizing epithelial cell yield and differentiation for experimental use. While we failed to expand MTECs using commercially available media used for expansion of human airway epithelial cells, we successfully expanded MTECs in KFSM medium, which also prevented the outgrowth of fibroblasts, most likely due to its low calcium level [55,56]. Compared to animal models, the ALI in vitro culture with in vitro expanded MTECs is more cost-effective, time-efficient, easier to perform, and it reduces animal suffering. As a physiologically relevant in vitro model, it supports the 3R principle by enabling the generation of a large number of samples from a minimal number of animals, thus reducing animal use and improving reproducibility [14,57]. It also has the potential to provide mechanistic insights into the immune mechanisms of pulmonary allergy at the airway epithelial interface.

Pulmonary diseases such as allergic asthma affect over 300 million people worldwide, with a rising prevalence and increasing economic, social and healthcare system burden. Thus, there is an urgent need to better understand the mechanisms driving allergen sensitization in the upper airways. Inhaled allergens primarily interact with and activate airway epithelial cells, which subsequently release alarmins to activate dendritic cells and ILC2 cells [58]. ALI cultures closely mimic the in vivo airway epithelium by replicating key structural and functional features, including tight junction formation, ciliated and goblet cell differentiation and a physiologically relevant apical air-exposed interface. These characteristics make the murine ALI model particularly suited for studying epithelial responses to environmental stimuli and innate immune processes. To mimic the initial allergen encounter of MTECs, we designed a compact, autoclavable 3D-printable deposition device, which is connected to a commonly used nebulizer and allows for precise and reproducible aerosol deposition into a single TC insert. Previously, purpose-made deposition chambers have been designed and used to deliver precise dosage of drugs using machined polyoxymethylene (POM) [32] or hydrogel nanoparticles using a Carbon M1 3D-printing platform [59]. However, both approaches face limitations in accessibility due to their reliance on costly equipment: machined POM components require precision tools and expertise, while industrial-grade 3D-printing platforms like Carbon M1 involve significant upfront investment and operational costs, which may restrict their adoption in resource-constrained research settings. Furthermore, while POM has been shown to be relatively safe for cell culture application [60], the proprietary resin used by Sudduth et al. [59] is classified as prototyping resin, with no information regarding its cytotoxicity. In contrast, the Bio-Med resin employed in our study offers a cost-effective and accessible alternative, specifically designed for compatibility with hobby-grade resin 3D printers. This material is commercially available at a fraction of the cost of industrial-grade alternatives, eliminating the need for specialized machining tools or high-end printing platforms. Critically, the resin has undergone rigorous biocompatibility testing, meeting ISO 10993-5 (cytotoxicity), ISO 10993-10 (irritation and skin sensitization) and ISO 10993-23 (genotoxicity) standards, ensuring its safety for biomedical applications (Available online: https://www.liqcreate.com/product/bio-med-clear-biocompatible-resin/ accessed on 9 October 2025).

Here, we used the deposition device to nebulize clinically highly relevant aeroallergen HDM, affecting 1–2% of the global population [61]. HDM proteases can activate the airway complement system, leading to the generation of anaphylatoxins C3a and C5a, which contribute and regulate several immune responses in HDM-driven allergic asthma. HDM-derived proteases can directly cleave C3 and C5 to produce C3a and C5a, as demonstrated in purified systems, and these activities are also observed in HDM extracts [62]. In murine models of HDM-induced allergic asthma, elevated C5a levels in bronchoalveolar lavage (BAL) fluid correlate with Th2 cell differentiation and airway hyper-responsiveness (AHR), which are attenuated by C5 blockade [63]. Complement activation further influences eosinophil trafficking, as inflammatory eosinophils (iEOS) express higher levels of C5aR1, and C5aR1 deficiency reduces pulmonary iEOS recruitment and degranulation, impairing AHR [21]. In humans with mild asthma, segmental allergen challenge induces significant increases in BAL C3a and C5a levels 24 h after challenge, correlating with eosinophil and neutrophil infiltration [20]. Additionally, C5a signaling, through its receptor C5aR2, modulates dendritic-cell-mediated Th2 and Th17 responses in HDM-exposed mice, highlighting the dual roles of complement in shaping adaptive immunity [64]. While some studies report no significant complement activation during early or late bronchial reactions in humans [65], the collective evidence underscores the pathogenic role of HDM-driven complement activation in allergic asthma, mediated via protease-induced anaphylatoxin generation and receptor-dependent immune cell interactions.

Reports regarding the production of C3 and C5 from primary mouse AE cells are scarce [26]. Here, we show the expression of C3 and C5 and their cleavage into C3a and C5a in functional primary AE cells, as well as the dynamic expression of C3aR and C5aR1 in response to HDM stimulation. Of note, AE cells produced similar levels of C5 and C3, independent of HDM exposure. However, while significant amounts of C3 were secreted by the AE cells in response to HDM nebulization, this was not the case for C5. Also, given the molecular mass of mouse C3 (170 kDa) and C3a (10 kDa), our results suggest that most of the C3 is converted to C3a in response to HDM, both within the AE cells and after secretion. In contrast, this is not the case for C5a. In line with previous reports demonstrating HDM-protease-mediated cleavage of C3 into C3a, we found that HMD, but not HI-HDM, strongly cleaved C3 into C3a, suggesting that such proteases drive C3a generation from AE-secreted C3. The functional impact of this differential extracellular release of C3/C5 and the subsequent generation of C3a/C5a needs to be evaluated in follow-up studies.

Additionally, the upregulation of TSLP aligns with its well-established role as a key factor of allergic airway inflammation. Despite the absence of immune cells, the presence of TSLP in both lysates and the SN indicates active synthesis and subsequent secretion by the ALI cultures, particularly at 72 h. This finding supports previous studies showing that TSLP induction often follows initial inflammatory signals [66]. IL-33 was highly expressed at baseline, consistent with its role as a constitutive nuclear alarmin, poised to respond immediately to tissue damage or stress. Its decline over time in lysates indicates active release extracellularly. The absence of measurable IL-33 in the SN is likely due to its rapid oxidation and the formation of disulfide bridges in the culture medium, which limits its biological activity and prevents in vivo enhancement of inflammation when this mechanism fails [67]. IL-25 showed a delayed but significant increase in lysates at 72 h. Although typically associated with immune cell activation, IL-25 is also produced by epithelial cells and has been shown to induce angiogenesis [68], enhance endothelial VEGF expression [68] and promote airway remodeling through ECM deposition [69].

These results highlight the ability of murine ALI cultures to reproduce physiologically relevant epithelial behaviors, such as barrier disruption and immune modulation, under controlled experimental conditions. Additionally, murine ALI cultures offer several practical and methodological advantages over other in vitro systems, such as organoids. The ease of setup and maintenance, requiring minimal specialized tools, makes ALI cultures more accessible for routine studies. Moreover, the air-exposed apical surface uniquely positions ALI cultures as a physiologically relevant model for investigating respiratory exposure to airborne allergens, pathogens and pollutants. Compared to organoids, ALI cultures also facilitate direct and uniform application of stimuli, such as nebulized HDM, and allow precise monitoring of epithelial barrier integrity through measurements like TEER. The custom data logger for the Millicell-ERS2 system minimizes variability caused by probe submersion angle and temperature fluctuations. Additionally, the custom logger saves the operator time by logging the data into a retrievable text file and enhances accuracy by averaging two hundred readings per second.

In summary, the optimized expansion, propagation and differentiation media ensured the successful creation and maintenance of murine ALI cultures, addressing common challenges associated with cell yield and fibroblast contamination. This refinement enhanced the reproducibility and scalability of the model, making it a valuable platform for mechanistic studies of epithelial immune responses. Our findings demonstrate that murine ALI cultures effectively recapitulate key aspects of epithelial-driven immune responses relevant to allergic airway inflammation. The dynamic expression and cleavage of complement components C3 and C5, along with the upregulation of their receptors, highlight early innate immune activation in response to HDM stimulation. The dynamic and time-dependent changes in TSLP, IL-33 and IL-25 underscore the power of ALI cultures to mimic in vivo inflammatory response of airway epithelium even in the absence of immune cells. Despite its strengths, the murine ALI model remains a simplified system that lacks the multicellular complexity of in vivo environments. The absence of co-cultured immune cells, such as macrophages or dendritic cells, limits the ability to study the cross-talk between the epithelium and the immune system. Incorporating such cell types in future iterations of the model could provide deeper insights into the dynamic interplay between epithelial cells and innate immune responses.

Furthermore, while our study focused on C3 and C5 expression, the generation of anaphylatoxins C3a and C5a and the expression of their cognate C3aR and C5aR1, complement activation and signaling involve a broader network of proteins and pathways. Future studies could expand on this work by profiling additional components of the complement cascade, propagating epithelial cells from complement or complement-receptor-deficient mice and exploring their interactions with other immune pathways under both steady-state and stimulated conditions.

## 5. Conclusions

The murine ALI culture model represents a methodologically sound and physiologically relevant platform for studying the innate immune system and complement biology of the airway epithelium. Its ability to replicate key in vivo epithelial functions, combined with practical advantages, such as scalability and accessibility, makes it an invaluable tool for respiratory immunity research. The model’s utility is further enhanced by its adaptability to diverse experimental conditions, including the precise application of airborne stimuli and monitoring of epithelial barrier integrity. Continued optimization of the system, including co-culture with immune cells, promises to broaden its applicability and deepen our understanding of airway immunity and inflammation.

## Data Availability

The datasets used and/or analyzed in the current study are available from the corresponding author upon reasonable request.

## Supplementary Materials

The following supporting information can be downloaded at: https://www.mdpi.com/article/10.3390/cells14201598/s1, Document S1: Schematics and code for the ERS2 data logger; File S1: Part1_ERS2 case logger box & Part2_ERS2 case logger lid; File S2: Chamber technical sketch & Chamber 3D Sketch; Video S1: Digital microscopy of moving cilia.

## Abbreviations

The following abbreviations are used in this manuscript:

AE	Airway epithelium
PAMPs	Pathogen-associated molecular patterns
ALI	Air–liquid interface
HDM	House dust mite
TEER	Transepithelial electrical resistance
BPE	Bovine pituitary extract
ITS-G	Insulin–transferrin–selenium
RA	Retinoic acid
TC	Tissue culture
RT	Room temperature
SEM	Standard error of the mean
ANOVA	Analysis of variance
MTECs	Murine tracheal epithelial cells
SN	Supernatant
TSLP	Thymic stromal lymphopoietin
POM	Polyoxymethylene
BAL	Bronchoalveolar lavage
AHR	Airway hyper-responsiveness
iEOS	Inflammatory eosinophils
