# Supplementary material for: House Dust Mite Nebulization Drives Alarmin and Complement Activation in a Murine Tracheal Air–Liquid Interface Culture System
Source: Cells. 2025 Oct 14;14(20):1598. doi: 10.3390/cells14201598 (PMC12563611; doi:10.3390/cells14201598)
Supplement: Supplementary file 1 [file cells-14-01598-s001.zip › File S2 - chamber technical sketch.pdf]

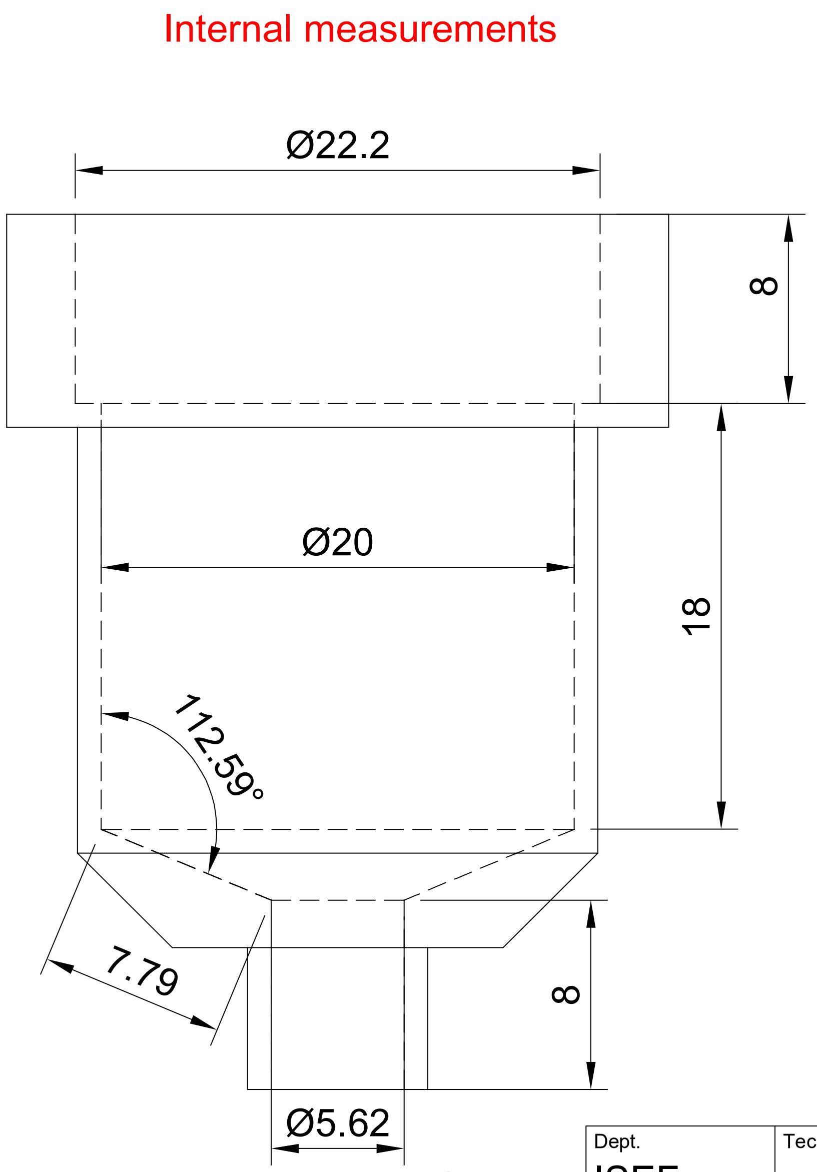

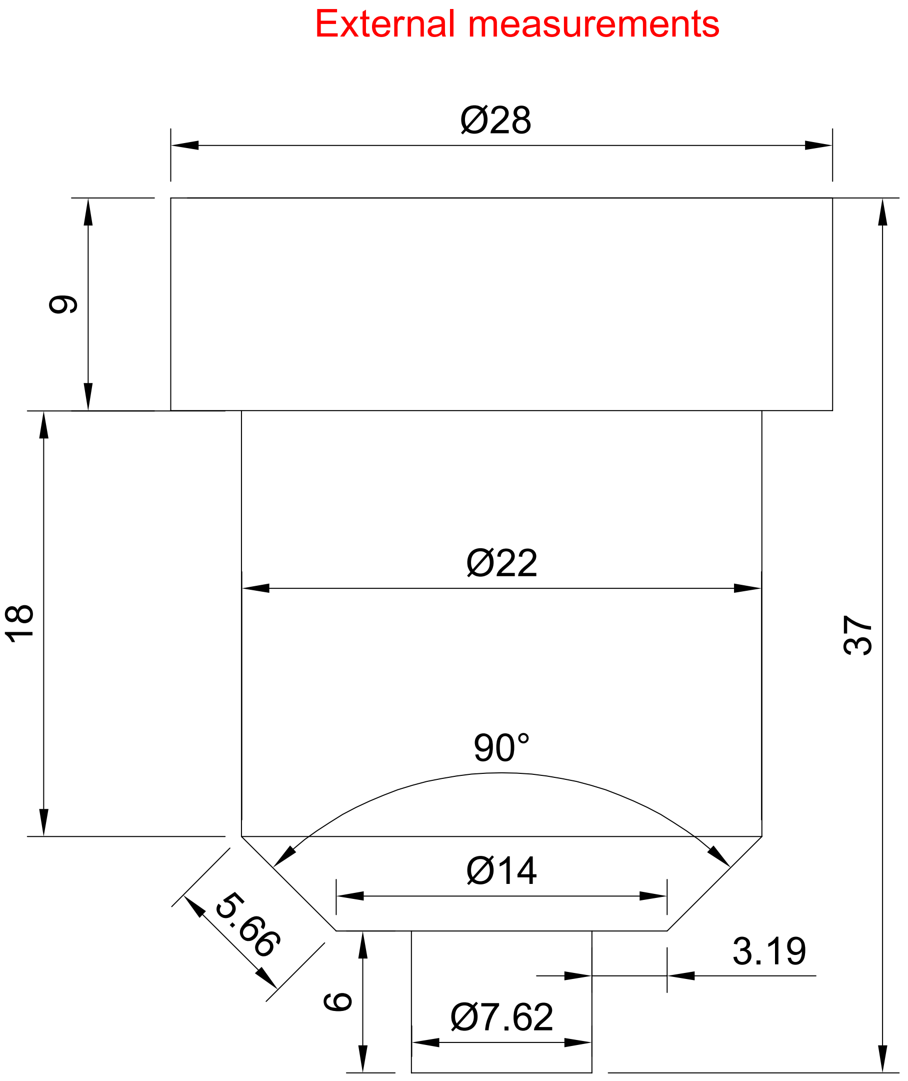

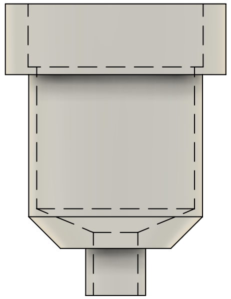

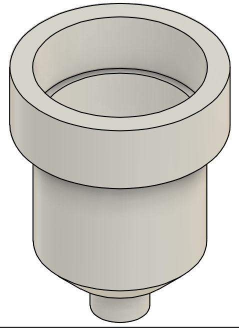

Dept.	Technical reference	Created by	Approved by
ISEF		Janti Haj Ahmad	
		Document type	Document status
		Title: ALI-Deposition Chamber	DWG No.
			Rev. / Date of issue / Sheet: 1/1
