# Supplementary material for: House Dust Mite Nebulization Drives Alarmin and Complement Activation in a Murine Tracheal Air–Liquid Interface Culture System
Source: Cells. 2025 Oct 14;14(20):1598. doi: 10.3390/cells14201598 (PMC12563611; doi:10.3390/cells14201598)
Supplement: Supplementary file 1 [file cells-14-01598-s001.zip › Document S1.pdf]

Schematic for EVOM2 data logger:

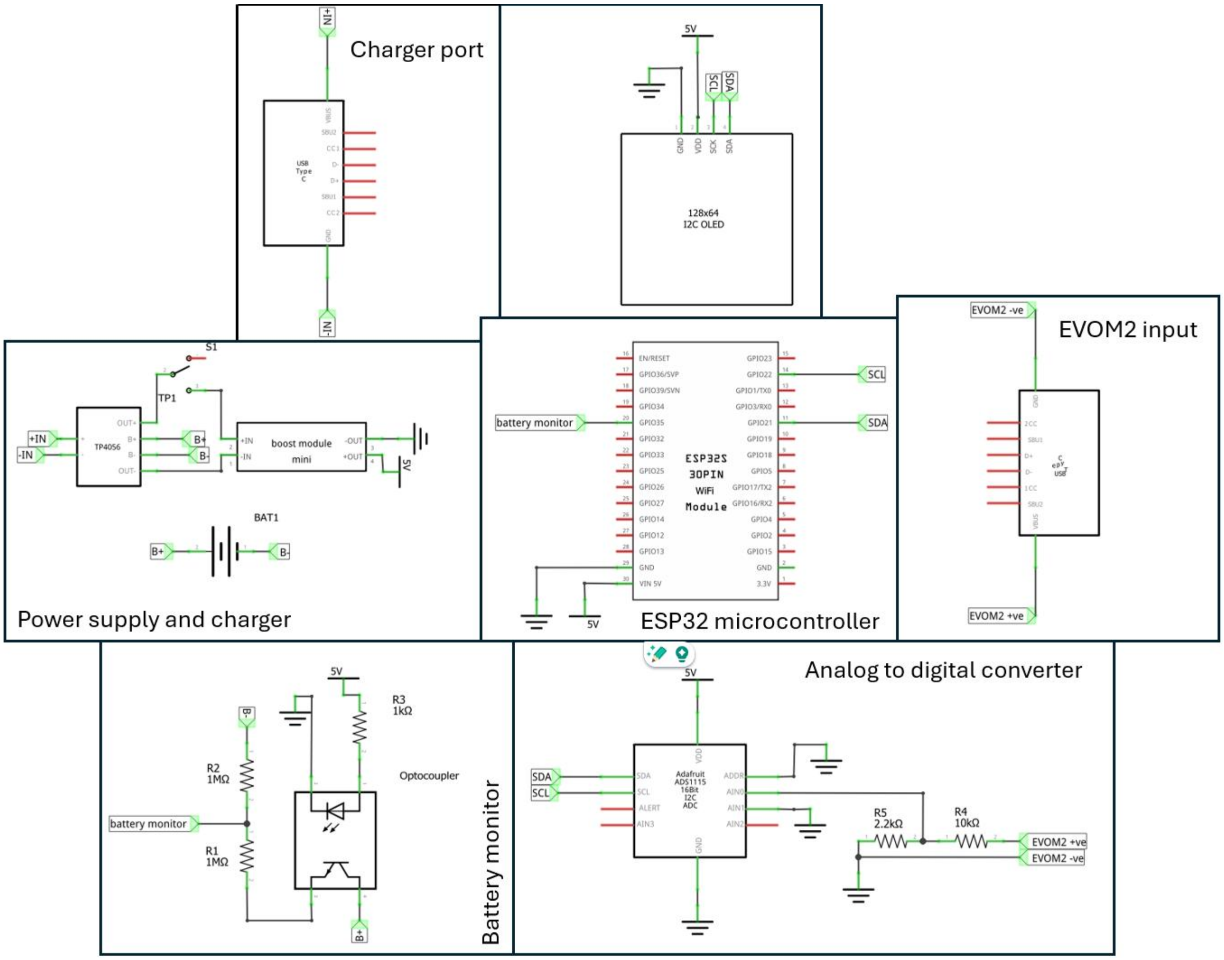

List of materials:

- X1 ESP32 30 pins microcontroller
- X2 female USB-C ports
- X2 1MΩ resistors
- X1 PC817 Optocoupler
- X1 1kΩ resistor
- X1 3.7v Lipo battery
- X1 TP4056
- X1 5V boost converter
- X1 switch
- X1 SSD1306 OLED screen
- X1 ADS1115
- X1 2.2kΩ resistor
- X1 10kΩ resistor

ESP32 microcontroller code (Arduino IDE)

```
#include <WiFi.h>
#include <SPIFFS.h>
#include <WebServer.h>
#include "Adafruit_ADS1X15.h"  /* ADS115 16-bit ADC library */
#include "Adafruit_GFX.h"      // Core graphics library
#include "Adafruit_SSD1306.h"  // SSD1306 OLED display library

// WiFi credentials
const char* ssid = "EVOM2 Data Logger";  // Set your desired AP SSID
const char* password = "123456789";      // Set your desired AP password

// Create server object
WebServer server;

// OLED and ADC objects
#define OLED_RESET -1  // OLED reset pin (not used)
Adafruit_SSD1306 display(OLED_RESET);  // OLED display object
Adafruit_ADS1115 ads;  // ADC object

// Battery monitoring pin (use an available ADC pin)
#define BATTERY_PIN 35  // ADC pin for battery voltage reading

// Voltage divider values (adjust based on your resistors)
const float R1 = 1000000.0; // Resistor 1 value (Ohms)
const float R2 = 1000000.0; // Resistor 2 value (Ohms)

// Maximum battery voltage (adjust based on battery type)
const float MAX_BATTERY_VOLTAGE = 4.2;  // For LiPo battery

// TEER calculation variables
float teer = 0; // Initialize TEER value to 0
float adsMultiplier = 0.125F;   // 1 bit = 0.125 mV for GAIN_ONE mode
float minTeerThreshold = 10.0;  // Minimum TEER value (Ohms) required to start
logging (can be changed)

// Function to read ADC
float readADC() {
    float rawADC = 0;    // Read ADC 200 times (can be increased) to reduce noise
and ADC imperfections
    for (int i = 0; i < 200; i++) {
        rawADC += ads.readADC_Differential_0_1();  // Read differential voltage
between A0 and A1
    }
    float averageADC = rawADC / 200.0; // Get the average ADC value
```

```
    // Convert ADC value to voltage using the ADS1115's resolution (0.125 mV per
bit for GAIN_ONE)
    float resistance = averageADC * adsMultiplier;  // adsMultiplier is 0.125
mV/bit for GAIN_ONE

    // Compensating for the voltage divider (4.74 can change based on the input
voltage compared to the voltage input into A0 pin of ADS1115)
    float TEER = resistance * 4.74;

    return TEER;  // Return the calculated value
}

// Function to read battery voltage
float readBatteryVoltage() {
    int rawValue = analogRead(BATTERY_PIN);  // Read the raw ADC value
    float voltage = rawValue * (3.3 / 4095.0);  // Convert to voltage (ESP32 has
12-bit ADC resolution, hence 4095)
    float batteryVoltage = voltage / (R2 / (R1 + R2));  // Adjust for voltage
divider

    return batteryVoltage;
}

// Function to get battery percentage
float getBatteryPercentage(float voltage) {
    return (voltage / MAX_BATTERY_VOLTAGE) * 100.0;  // Calculate percentage
based on max battery voltage
}

// Function to display data on OLED
void displayData(float teer, String message, float batteryVoltage) {
    display.setTextColor(SSD1306_WHITE); // Set text color
    display.setTextSize(1);

    // Calculate center position for the message
    int16_t xPos = (128 - (message.length() * 6)) / 2;  // Each character is
approximately 6 pixels wide
    int16_t yPos = 0;  // Message at the top

    // Draw the message
    display.setCursor(xPos, yPos);
    display.print(message);

    // Create string for the TEER value
    String teerStr = String(teer) + " Ohms";
```

```
    xPos = (128 - (teerStr.length() * 6)) / 2;  // Calculate the center position
for the TEER value
    yPos = 20;  // Adjust vertical position

    display.setCursor(xPos, yPos);
    display.print(teerStr);  // Display TEER value

    // Display battery percentage or voltage
    String batteryStr = "Battery: " + String(batteryVoltage, 2) + "V";
    yPos = 40;  // Adjust vertical position for battery status
    display.setCursor(0, yPos);
    display.print(batteryStr);  // Display battery voltage

    display.display();  // Refresh the display with the new content
}

// Serve homepage with download and delete options
void handleRoot() {
    String page = "<html><body><h1>File Management</h1>";
    page += "<p><a href='/teer_log.txt' download>Download teer_log.txt</a></p>";
    page += "<p><a href='/delete'>Delete teer_log.txt</a></p>";
    page += "</body></html>";

    server.send(200, "text/html", page);
}

// Handle the file download request
void handleDownload() {
    File file = SPIFFS.open("/teer_log.txt", "r");
    if (!file) {
        server.send(404, "text/plain", "File Not Found");
        return;
    }

    server.streamFile(file, "text/plain");
    file.close();
}

// Handle the delete request
void handleDelete() {
    if (SPIFFS.remove("/teer_log.txt")) {
        server.send(200, "text/plain", "File deleted successfully");
    } else {
        server.send(404, "text/plain", "File Not Found");
    }
```

```
}

// Write some initial data to the log file
void writeLogFile(String data) {
    File file = SPIFFS.open("/teer_log.txt", "a");  // Open the file in append 
mode
    if (!file) {
        Serial.println("Failed to open file for writing");
        return;
    }
    file.println(data);  // Write the data to the file
    file.close();
    Serial.println("Data written to log file: " + data);
}

void setup() {
    Serial.begin(115200);

    // Set up the ESP32 as an Access Point with a static IP
    IPAddress local_ip(192, 168, 1, 1);
    IPAddress gateway(192, 168, 1, 1);
    IPAddress subnet(255, 255, 255, 0);

    WiFi.softAPConfig(local_ip, gateway, subnet);
    WiFi.softAP(ssid, password);
    Serial.println("Access Point started");
    Serial.print("IP Address: ");
    Serial.println(WiFi.softAPIP()); // Print the IP address of the AP

    // Initialize SPIFFS
    if (!SPIFFS.begin(true)) {
        Serial.println("Failed to mount SPIFFS");
        return;
    }
    Serial.println("SPIFFS mounted successfully");

    // Write initial data to teer_log.txt
    writeLogFile("TEER Measurement Log");
    writeLogFile("====================");

    // Set up web server routes
    server.on("/", HTTP_GET, handleRoot);
    server.on("/teer_log.txt", HTTP_GET, handleDownload);
    server.on("/delete", HTTP_GET, handleDelete);

    // Initialize OLED display
```

```
    display.begin(SSD1306_SWITCHCAPVCC, 0x3C);
    ads.setGain(GAIN_ONE); /* Select range and precision of ADC. */
    ads.begin();

    Serial.println("Initialization complete");
    displayData(teer, "Init Done", readBatteryVoltage());
    delay(2000);

    server.begin();
    Serial.println("HTTP server started");
}

void loop() {
    static bool firstRun = true;
    static bool loggingStarted = false;
    static unsigned long lastLogTime = 0;  // Timer for logging
    static unsigned long lastDisplayTime = 0;  // Timer for OLED display
    const unsigned long logInterval = 500;  // Log data time
    const unsigned long displayInterval = 500;  // Update OLED time

    if (firstRun) {
        delay(1000);  // Initial delay before starting to check TEER value
        firstRun = false;
    }

    server.handleClient();  // Handle incoming client requests

    // Read the battery voltage
    float batteryVoltage = analogRead(BATTERY_PIN) * (3.3 / 4095.0) * 2.14;  //
Adjust for voltage divider (in this case 2.14). R1 is 1M and R2 is 1M ratio
should be 2.0, since the resistors are not perfect, real world ration is 2.14
    float batteryPercentage = (batteryVoltage - 3.0) / (4.15 - 3.0) * 100;
    if (batteryPercentage > 100) batteryPercentage = 100;
    if (batteryPercentage < 0) batteryPercentage = 0;

    // Read TEER value from ADC
    float r = readADC();  // Call readADC to get the resistance value

    // Log data every 'logInterval' milliseconds (5 seconds in this case)
    if (millis() - lastLogTime >= logInterval) {
        lastLogTime = millis();  // Reset timer
        if (r >= minTeerThreshold && !loggingStarted) {
            loggingStarted = true;  // Start logging if threshold is met
            Serial.println("TEER threshold met, starting logging...");
        }
```

```
        // If logging started, log the data
        if (loggingStarted) {
            // Write TEER reading to log file
            String logData = "TEER: " + String(r, 2) + " Ohms at " +
String(millis()) + " ms";
            writeLogFile(logData);  // Append the data to the file
            Serial.println("Logged data: " + logData);  // Debugging message
        }
    }

    // Update OLED display every 'displayInterval' milliseconds (1 second in this
case)
    if (millis() - lastDisplayTime >= displayInterval) {
        lastDisplayTime = millis();  // Reset timer
        // Clear the display before updating it
        display.clearDisplay();

        // Display battery info at the top
        display.setCursor(0, 0);  // Set cursor to top of the screen
        display.setTextSize(1);    // Set text size to small
        display.print("Battery: ");
        display.print(batteryVoltage, 2);  // Show battery voltage with 2 decimal
places
        display.print("V (");
        display.print(batteryPercentage, 0);  // Show battery percentage as
integer
        display.print("%)");

        // Display TEER value below the battery info
        display.setCursor(0, 20);  // Adjust vertical position for TEER display
        display.setTextSize(1);    // Keep text size consistent
        display.print("TEER: ");
        display.print(r, 2);  // Display TEER value with 2 decimal places
        display.print(" Ohms");

        display.display();  // Refresh the display with the new content
    }
}
```
